# A potential regulatory region near the EDN3 gene may control both harness racing performance and coat color variation in horses

**DOI:** 10.14814/phy2.13700

**Published:** 2018-05-24

**Authors:** Kim Jäderkvist Fegraeus, Brandon D. Velie, Jeanette Axelsson, Rachel Ang, Natasha A. Hamilton, Leif Andersson, Jennifer R. S. Meadows, Gabriella Lindgren

**Affiliations:** ^1^ Department of Animal Breeding & Genetics Swedish University of Agricultural Sciences Uppsala Sweden; ^2^ Faculty of Science University of Sydney Sydney Australia; ^3^ Department of Medical Biochemistry and Microbiology Science for Life Laboratory Uppsala University Uppsala Sweden; ^4^ Department of Veterinary Integrative Biosciences Texas A&M University College Station Texas

**Keywords:** Coldblooded trotter, Fixation Index, Fst

## Abstract

The Swedish‐Norwegian Coldblooded trotter and the heavier North‐Swedish draught horse both descend from the North‐Swedish horse, but the Coldblooded trotters have been selected for racing performance while the North‐Swedish draught horse is mainly used for agricultural and forestry work. By comparing the genomes of Coldblooded trotters, North‐Swedish draught horses and Standardbreds for a large number of single‐nucleotide polymorphisms (SNPs), the aim of the study was to identify genetic regions that may be under selection for racing performance. We hypothesized that the selection for racing performance, in combination with unauthorized crossbreeding of Coldblooded trotters and Standardbreds, has created regions in the genome where the Coldblooded trotters and Standardbreds are similar, but differ from the North‐Swedish draught horse. A fixation index (Fst) analysis was performed and sliding window Delta Fst values were calculated across the three breeds. Five windows, where the average Fst between Coldblooded trotters and Standardbreds was low and the average Fst between Coldblooded trotters and North‐Swedish draught horses was high, were selected for further investigation. Associations between the most highly ranked SNPs and harness racing performance were analyzed in 400 raced Coldblooded trotters with race records. One SNP showed a significant association with racing performance, with the CC genotype appearing to be negatively associated. The SNP identified was genotyped in 1915 horses of 18 different breeds. The frequency of the TT genotype was high in breeds typically used for racing and show jumping while the frequency of the CC genotype was high in most pony breeds and draught horses. The closest gene in this region was the *Endothelin3* gene (*EDN3*), a gene mainly involved in melanocyte and enteric neuron development. Both functional genetic and physiological studies are needed to fully understand the possible impacts of the gene on racing performance.

## Introduction

Descending from the North‐Swedish horse, the Coldblooded trotter is a unique racehorse breed with a draught horse origin. During the 19th and the first part of the 20th century most North‐Swedish horses were used for forestry and agricultural work. However, horse racing has fascinated humans for hundreds of years and many of the North‐Swedish horses were not only used for work but also for racing. The first official harness race with North‐Swedish horses took place already in the early 1800s. When interest in harness racing grew breeders began to select for faster and more sustainable horses. While many of the horses were used for both working and racing, the intensive selection for racing performance traits started to negatively influence the breeding of good working horses. Therefore, in 1964, the decision was made to divide the North‐Swedish horse into two different breeds: the North‐Swedish draught horse and the lighter Coldblooded trotter (Bohlin and Rönningen 1975). During the last 50–60 years there has been intense selection for high‐performing Coldblooded trotters and the performance of the breed has significantly improved (Árnason et al. 1989; Árnason 2001; Thiruvenkadan et al. 2009). Also, although not allowed, it is well known that before parentage testing was introduced in 1969, Coldblooded trotters were crossbred with the faster, lighter, and more energetic Standardbred to create better racehorses.

One important breeding goal of the Coldblooded trotter is to maintain the light draught horse appearance of the breed. Consequently, the Coldblooded trotters and the North‐Swedish draught horses display many phenotypic similarities. Due to their common origin, the genetic makeup of the two breeds is also very similar. However, due to the disparate selection of the breeds there are regions in the genome where the two breeds differ. Racing under the same conditions and regulations, both Coldblooded trotters and Standardbreds have been selected for genetic variants with a positive impact on racing performance. Therefore, our hypothesis is that these two breeds share a number of genetic variants that differ from the variants observed in North‐Swedish draught horses. As such, by comparing the allele frequencies for a large number of single‐nucleotide polymorphisms (SNPs) between Coldblooded trotters, North‐Swedish draught horses and Standardbreds, the aim of the study was to identify genetic regions that are under selection for harness racing performance. These regions may for example contain genes influencing energy metabolism, muscle composition or temperament, as these are all examples of traits where the Coldblooded trotters are similar to Standardbreds but different from the North‐Swedish draught horses. Identifying novel genes important for performance is not only of value for the racing industry. Genes that take part in the regulation of energy metabolism and other biological processes that impact racing performance may also shed light on metabolic defects and diseases in horses as well as in other species.

## Material and Methods

The study was divided into three different parts: (1) a Delta fixation index (Fst) analysis, (2) a SNP association analysis, (3) genotyping of the top‐SNP identified in the Delta Fst analysis in a variety of breeds.

### Part i: Delta Fst analysis

#### Horse material

In total, 42 horses (11 Coldblooded trotters, 19 North‐Swedish Draught horses, and 12 Standardbreds) were included in part i. The two trotting breeds were elite performing horses. They were selected based on Estimated Breeding Value (EBV) and pedigree, to only include horses that had been bred for racing, and to avoid including horses with the same parents or grandparents. The Identical By Descent (IBD) value was calculated in PLINK using the –genome command, and the threshold was set to maximum 0.25 (Purcell et al. 2007). The North‐Swedish draught horses were all approved breeding stallions. The horses were born between 1986 and 2001 (Coldblooded trotters), 1993 and 2000 (Standardbreds), and 1988 and 2007 (North‐Swedish draught horses).

### DNA extraction, genotyping and quality control

DNA was extracted from 350 *μ*L of blood using the Qiasymphony instrument (Qiagen, Hilden, Germany). The DNA samples were genotyped on one of two different Illumina SNP50 Genotyping BeadChips, one that contained 54,602 SNPs (Coldblooded trotters, *n* = 7; Standardbreds, *n* = 12; North‐Swedish draught horses, *n* = 5) and one that contained 57,165 SNPs (Coldblooded trotters, *n* = 4; Standardbreds, *n* = 7; North‐Swedish draught horses, *n* = 7). The two datasets were merged and quality control (QC) was performed in PLINK (Purcell et al. 2007). For the calculation of Fst between the breeds, two new datasets were created: set A including Coldblooded trotters and Standardbreds and set B including Coldblooded trotters together with the North‐Swedish draught horses. QC was performed for each dataset, excluding SNPs with a genotype call lower than 99% (*n*_A _= 11,763, *n*_B _= 13,125) or a minor allele frequency (maf) <0.00001 (*n*_A _= 4862, *n*_B _= 6535). In addition, SNPs that were not in common between the breeds (*n*_A _= 4695, *n*_B _= 1660) and X‐chromosome SNPs (*n*_A _= 1434, *n*_B _= 1434) were excluded from the analyses. In total, 37,246 SNPs remained and were included in the analyses.

### Statistical analysis

The statistical analyses were performed in PLINK and the software program for statistical computing R (Purcell et al. 2007; R Development Core Team, 2016). A sliding window Fst analysis was performed across all breeds. The Fst between the breeds was calculated for each SNP according to Wrights definition; var(p)/(p(1‐p)), where p is the average minor allele frequency for the two breeds compared (Brown 1970). The average Delta Fst was calculated from windows of 5 SNPs, using ΔFST = FST[Set B] ‐ FST[Set A]. The five top windows, where the Fst in set A was low, and the Fst in set B was high (i.e., where the Coldblooded trotters and Standardbreds were genetically similar, but together differed from the North‐Swedish draught horses) were selected for further investigation for association with harness racing performance.

### Part ii: Association analysis of the highest ranked SNPs with racing performance in 400 Coldblooded trotters

#### Horse material

The association between the highest ranked SNPs and overall career racing performance was investigated in about 400 raced Coldblooded trotters born between 2000 and 2009.

##### Phenotype information

Racing performance data for the years 2003–2015 was provided by the Swedish Trotting Association. The following performance traits were analyzed:

*Rankings*: The number of wins was calculated as the total number of times a horse finished a race in first place. The number of placings was calculated as the total number of times a horse finished a race in first, second or third place.

*Race times:* For race time records two different starting methods were included in the study: autostart and voltstart (Thiruvenkadan et al. 2009). The best race times for each horse were defined as the lowest average time (in seconds) per kilometer, for each starting method.

*Earnings:* The majority of the earnings provided were in Swedish currency (SEK), but the earnings for Norwegian trotters were in Norwegian currency (NOK). In order to set all earnings to Swedish currency an average exchange rate was calculated (*μ *= 0.95) for the years 2003–2015 and multiplied with the Norwegian earnings (Valuta, 9999). Earnings per start were calculated as the amount of prize money earned per start.

### Genotyping

Five windows with the highest Delta Fst values (window) were selected from the Fst analysis. From those, the SNP with the highest single Delta Fst value was selected and its association with racing performance in Coldblooded trotters was investigated. All Coldblooded trotters had previously been genotyped on the 670K Axiom Equine Genotyping array (Jäderkvist Fegraeus et al. 2017).

### Statistical analyses

#### Single SNP analyses

The statistical analyses were performed in R (R Development Core Team, 2016). Summary statistics for each performance trait were calculated based on raw values for the whole career. To obtain normally distributed values, earnings and best race times were transformed according to two different previously published formulas: ln(earnings +1 000) and ln(racing time −68.2) (Árnason 1994). All other non‐normally distributed traits were log10‐transformed. Horses with no time records using autostart or voltstart were excluded from all analyses concerning the corresponding starting method. Each performance trait was analyzed using linear models. All models included fixed effects of sex, age and country of registration. Number of starts was included when applicable. In addition, the genotype for the SNP 23:22999655 in the Doublesex And Mab‐3 Related Transcription Factor 3 gene (*DMRT3*) was included as a covariate due to previous studies having shown a major impact of this gene on harness racing performance (Andersson et al. 2012; Jäderkvist et al. 2014; Jäderkvist Fegraeus et al. 2017).

#### Haplotype analyses

If a significant association was identified between a SNP and racing performance, haplotype analysis was performed using the surrounding SNPs that were in LD with the SNP identified. A GLM regression analysis was performed using the haplo.stats package in R, to test for association between haplotype and racing performance (Sinnwell and Schaid 2016). The model included the effects of *DMRT3* genotype, sex, age, and country of registration as well as number of starts, when applicable. Haplotypes with a frequency lower than 2% were considered rare and were not analyzed for association with performance.

### Part iii: Genotype frequency distribution for the SNPs significantly associated with racing performance, in 18 different horse breeds

#### Horse material

In total, 1915 horses of 18 different breeds were genotyped for the SNPs significantly associated with harness racing performance in part ii.

### Genotyping

The horses were either genotyped for the most significantly associated SNP with the StepOnePlus Real‐Time PCR System (Thermo Fisher) using a custom designed TaqMan SNP Genotyping Assay (Applied Biosystems) (*n* = 1553), or had previously been genotyped on the 670K Affymetrix Equine Genotyping Array (Exmoor pony, *n* = 271) (Velie et al. 2016) or the Illumina SNP70 Genotyping BeadChip (Thoroughbred, *n* = 91).

## Results

### Overall Fst between the three breeds

Based on 37,246 SNPs the average Fst values between the three breeds included in the Delta Fst analysis were: 0.082 (Coldblooded trotters vs. Standardbreds), 0.041 (Coldblooded trotters vs. North‐Swedish draught horses) and 0.088 (North‐Swedish draught horses vs. Standardbreds).

### Part i: Delta Fst analysis

**Figure 1:**
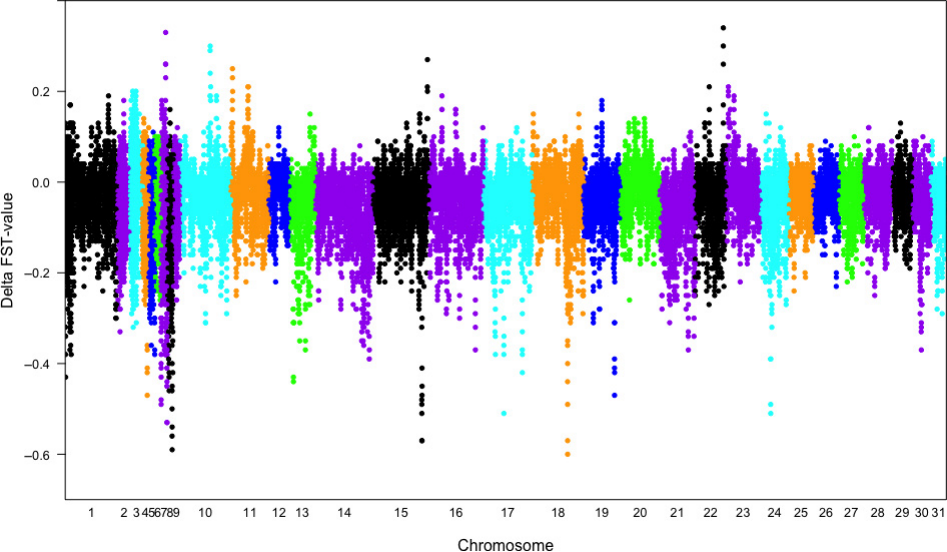
Delta Fst‐values (ΔFST = FST[Set B, Coldblooded trotters and Standardbreds] ‐ FST[Set A, Coldblooded trotters and North‐Swedish draught horses] based on sliding windows of 5 SNPs, in total 37,246 SNPs (ΔFST ranging from −1 to +1). A positive Delta Fst value means that the Fst for the SNP was low between Coldblooded trotters and Standardbreds but high between Coldblooded trotters and North‐Swedish draught horses. A negative Delta Fst value means that the Fst for the SNP was high between Coldblooded trotters and Standardbreds but low between Coldblooded trotters and North‐Swedish draught horses.

Five windows with the highest Delta Fst values were identified on different chromosomes (Figure 1). All the five windows had a Delta Fst value above 0.25. If two closely located windows had a similar Delta Fst value, only the window with the highest Delta Fst value was selected, as the SNPs were likely to be in linkage disequilibrium (LD). From each window one SNP with the highest single SNP Delta Fst value was selected for further investigation with harness racing performance. The allele frequencies for the top five SNPs identified are presented in Table 1 and include four intergenic (chr 7, 10, 11, 22) and one intronic (chr 15) SNP. The highest ranked window was located about 52 kb downstream of the *Endothelin 3* (*EDN3*) gene (chr22:45,674,895‐45,696,466) (Wade et al. 2009). The second window included the tsukushi (TSKU) gene (chr7:67,506,431‐67,507,477) and the third window was located 95.7 kb upstream of the F‐box and leucine‐rich repeat protein 4 (FBXL4) gene (chr10:50,098,114‐50,164,868). The fourth window, on chromosome 15, included two genes, allantoicase (ALLC) (chr15: 88,505,767‐88,529,774) and collectin subfamily member 11 gene (COLEC 11) (chr15:88,550,817‐88,582,392). The fifth window was located in the regulatory‐associated protein of MTOR complex 1 gene (RPTOR) (chr11:2,115,212‐2,506,974) (Wade et al. 2009).

**Table 1:** Allele frequencies for the top‐five SNPs identified in the Delta‐Fst analysis

SNP position	*n*	22:45,748,491		7:67,498,458		10:49,931,991		15:88,565,665		11:2,517,091	
Allele		T	C	T	C	G	A	C	T	T	G
Coldblooded trotter	11	0.80	0.20	0.06	0.94	0.22	0.78	0.95	0.05	0.91	0.09
North‐Swedish draught horse	19	0.15	0.85	0.74	0.26	0.90	0.10	0.36	0.64	0.34	0.66
Standardbred	12	0.75	0.25	0.25	0.75	0.25	0.75	0.91	0.09	0.75	0.25

### Part ii: Association analysis of the top markers and racing performance in 400 Coldblooded trotters

The allele frequencies for the five top SNPs in the raced Coldblooded trotters are presented in Table 2.

**Table 2:** Allele frequencies for the top‐five SNPs in Coldblooded trotters

Allele frequencies							
SNP position	*n*	T	C	A	G	T	G
22: 45,748,491	378	0.68	0.32	–	–	–	–
7: 67, 498,458	403	0.24	0.76	–	–	–	–
10: 49,431,991	386	–	–	0.70	0.30	–	–
15: 88,565,665	226	0.19	0.81	–	–	–	–
11: 2,517,091	400	–	–	–	–	0.84	0.16

### Single SNP analyses

Only the SNP g.22:45748491C>T showed significant associations with racing performance in Coldblooded trotters (six out of nine traits, *P* < 0.04, Table 3). For a complete summary of the performance statistics, see Table 4. Four SNPs in high LD with the most significant SNP (D′ = 0.97, *r* ^2^ = 0.92–0.94) also showed significant associations with racing performance (Tables 5, 6, 7, 8). The results were consistent with a recessive negative effect on racing performance of the haplotype associated with the C allele.

**Table 3:** Coldblooded trotter performance results for SNP g.22:45748491C>T

Genotype	TT (*n* = 106–173)		TC (*n* = 112–167)		CC (*n* = 15–38)		*P‐*values^b^		
Performance trait^a^	Mean	Median	Mean	Median	Mean	Median	TT vs. TC	TT vs. CC	TC vs. CC
No. of starts	37.6	29.0	37.7	26.0	23.1	18.0	0.94	**0.01**	**0.02**
No. of wins	4.1	2.0	4.1	2.0	1.9	1.0	0.72	**0.006**	**0.01**
No. of placings (1–3)	11.1	6.0	11.5	7.0	6.0	3.0	0.08	0.10	**0.007**
Wins (freq.)	0.10	0.07	0.09	0.07	0.06	0.02	0.28	0.84	0.39
Placings (freq.)	0.24	0.23	0.28	0.27	0.20	0.16	0.46	0.66	0.38
Earnings (SEK)^c^	235,900	81,000	249,300	102,200	97,280	42,290	0.12	0.27	**0.04**
Earnings/start (SEK)^c^	4,734	3,152	4,965	3,917	3,108	2,332	0.21	**0.02**	**0.003**
Time record voltstart (sec/km)	90.8	90.4	90.5	90.0	92.6	91.8	0.09	0.15	**0.01**
Time record autostart (sec/km)	88.8	88.6	88.8	88.6	90.2	90.4	0.95	0.31	0.33

a Transformed values were used for the analysis: log_10_, ln(earnings +1 000) and ln(race time −68.2).

b Linear model analyses were performed in R. Significant results (*P *≤* *0.05) in bold.

c SEK, Swedish Kronor.

**Table 4:** Summary statistics for SNP g.22:45748491C>T in Coldblooded trotters

Trait	TT (*n *= 106–173)						TC (*n *= 112–167)						CC (*n *= 15–38)					
	Min	Ist Q	Median	Mean	3rd Q	Max	Min	Ist Q	Median	Mean	3 rd Q	Max	Min	IstQ	Median	Mean	3 rd Q	Max
No. of starts	1.0	12.0	29.0	37.6	52.0	169.0	1.0	11.5	26.0	37.7	53.5	210.0	1.0	7.5	18.0	23.1	37.8	82.0
No. of wins	0.0	0.0	2.0	4.1	5.0	25.0	0.0	0.0	2.0	4.1	5.0	53.0	0.0	0.0	1.0	1.9	2.0	29.0
No. of placings	0.0	2.0	6.0	11.1	14.0	70.0	0.0	3.0	7.0	11.5	15.0	101.0	0.0	1.0	3.0	6.0	7.0	56.0
Wins (freq.)	0.00	0.00	0.07	0.10	0.14	1.00	0.00	0.00	0.07	0.09	0.14	0.75	0.00	0.00	0.02	0.06	0.09	0.40
Placings (freq.)	0.00	0.12	0.23	0.24	0.35	1.00	0.00	0.17	0.27	0.28	0.37	1.00	0.00	0.08	0.16	0.20	0.27	0.68
Earnings (SEK)	0	27 500	81000	235 900	229 000	2 416 000	0	33 400	102 200	249 300	260 700	3 189 000	0	15 880	42 290	97 280	106 200	1 198 000
Earnings/start (SEK)	0	1729	3 152	4 734	5 337	54 220	0	2 248	3 917	4 965	5 715	28 980	0	1 160	2 332	3 108	3 647	14 610
Time record Volt (sec/km)	81.40	88.48	90.40	90.77	93.05	104.40	82.40	87.88	89.95	90.45	92.72	106.90	83.90	89.90	91.80	92.64	94.82	105.70
Time record Auto (sec/km)	80.70	86.60	88.60	88.76	90.78	96.00	80.10	87.00	88.60	88.83	91.20	99.30	83.30	88.90	90.40	90.21	91.60	95.40

**Table 5:** Coldblooded trotter performance results for SNP g.22:45748082T>G

Genotype Performance trait^a^	GG (*n* = 117–189)			TG (*n* = 112–167)			TT (*n* = 19–43)			*P*‐values^b^		
	Mean	Median	SE	Mean	Median	SE	Mean	Median	SE	GGvsTG	GGvsTT	TGvsTT
No. of starts	37.2	29.0	2.0	37.3	24.0	2.2	25.5	17.0	2.1	0.74	**0.005**	**0.009**
No. of Wins	4.1	2.0	0.3	4.2	2.0	0.4	2.0	1.0	0.4	0.75	**0.01**	**0.02**
No. of Placings	10.9	6.0	0.8	11.6	7.0	0.9	6.6	3.0	0.8	0.12	0.30	**0.05**
Wins (freq.)	0.10	0.06	0.01	0.10	0.08	0.01	0.06	0.03	0.01	0.23	0.82	0.60
Placings (freq.)	0.24	0.23	0.01	0.28	0.27	0.01	0.23	0.19	0.02	0.43	0.70	0.91
Earnings (SEK)^c^	226,800	86,100	23,143	259,000	101,400	30,785	106,700	43,500	17,275	0.07	0.56	0.09
Earnings/start (SEK)^c^	4641	3133	387	5224	3944	344	3219	2,630	243	0.14	**0.04**	**0.003**
Time record voltstart (sec/km)	90.8	90.4	0.3	90.5	90.0	0.3	92.5	91.5	0.4	**0.05**	0.40	**0.04**
Time record autostart (sec/km)	88.9	88.9	0.2	88.7	88.6	0.2	89.6	89.9	0.3	0.37	0.25	0.11

a Transformed values were used for the analysis: log_10_, ln(earnings + 1 000) and ln(race time −68.2).

b Linear model analyses were performed in R. Significant results (*P *≤* *0.05) in bold.

c SEK, Swedish Kronor.

**Table 6:** Coldblooded trotter performance results for SNP g.22:45748586G>A

Genotype Performance trait^a^	AA (*n* = 115–183)			AG (*n* = 110–165)			GG (*n* = 20–44)			*P*‐value^b^		
	Mean	Median	SE	Mean	Median	SE	Mean	Median	SE	AAvsAG	AAvsGG	AGvsGG
No. of starts	37.6	29.0	2.0	37.0	24.0	2.2	25.4	18.0	2.1	0.41	**0.003**	**0.01**
No. of Wins	4.1	2.0	0.3	4.2	2.0	0.4	1.9	1.0	0.4	0.98	**0.008**	**0.009**
No. of Placings	11.0	6.0	0.8	11.6	7.0	0.9	6.5	3.0	0.8	**0.05**	0.24	**0.02**
Wins (freq.)	0.09	0.06	0.01	0.10	0.08	0.01	0.06	0.03	0.01	0.41	0.94	0.55
Placings (freq.)	0.24	0.23	0.01	0.28	0.27	0.01	0.22	0.19	0.02	0.20	0.76	0.60
Earnings (SEK)^c^	229,100	86,100	23,287	260,500	101,400	30,655	104,700	42,290	16,609	**0.05**	0.43	**0.04**
Earnings/start (SEK)^c^	4653	3133	389	5844	3951	342	3162	2594	235	0.20	**0.02**	**0.002**
Time record voltstart (sec)/km	90.8	90.4	0.3	90.5	90.0	0.3	92.5	91.5	0.4	**0.04**	0.35	**0.03**
Time record autostart (sec/km)	89.0	88.9	0.2	88.8	88.6	0.2	89.8	89.9	0.3	0.36	0.25	0.10

a Transformed values were used for the analysis: log_10_, ln(earnings +1 000) and ln(race time −68.2).

b Linear model analyses were performed in R. Significant results (*P *≤* *0.05) in bold.

c SEK, Swedish Kronor.

**Table 7:** Coldblooded trotter performance results for SNP g.22:45749526G>A

Genotype Performance trait^a^	AA (*n* = 117–189)			AG (*n* = 113–168)			GG (*n* = 20–44)			*P*‐value^b^		
	Mean	Median	SE	Mean	Median	SE	Mean	Median	SE	AAvsAG	AAvsGG	AGvsGG
No. of starts	37.2	29.0	2.0	37.3	24.5	2.2	25.4	18.0	2.1	0.78	**0.006**	**0.01**
No. of Wins	4.1	2.0	0.3	4.1	2.0	0.4	1.9	1.0	0.4	0.62	**0.006**	**0.02**
No. of Placings	10.9	6.0	0.8	11.6	7.0	0.9	6.5	3.0	0.8	0.12	0.21	**0.03**
Wins (freq.)	0.10	0.06	0.01	0.09	0.08	0.01	0.06	0.03	0.01	0.32	0.99	0.52
Placings (freq.)	0.24	0.23	0.01	0.28	0.27	0.01	0.22	0.19	0.02	0.42	0.88	0.72
Earnings (SEK)^c^	226,900	86,100	23,226	258,100	101,800	30,762	104,700	42,290	17,183	0.08	0.40	0.06
Earnings/start (SEK)^c^	4646	3133	388	5206	3938	344	3162	2594	243	0.16	0.03	**0.002**
Time record voltstart (sec/km)	90.8	90.4	0.3	90.5	90.0	0.3	92.5	91.5	0.4	0.07	0.35	**0.04**
Time record autostart (sec/km)	88.9	88.9	0.2	88.8	88.6	0.2	89.8	89.9	0.3	0.41	0.24	0.10

a Transformed values were used for the analysis: log_10_, ln(earnings + 1 000) and ln(race time −68.2).

b Linear model analyses were performed in R. Significant results (*P *≤* *0.05) in bold.

c SEK, Swedish Kronor.

**Table 8:** Coldblooded trotter performance results for SNP g.22:45749595G>A

Genotype	AA (*n* = 116–188)			AG (*n* = 112–167)			GG (*n* = 20–44)			*P*‐value^b^		
Performance trait^a^	Mean	Median	SE	Mean	Median	SE	Mean	Median	SE	AAvsAG	AAvsGG	AGvsGG
No. of starts	37.1	29.0	2.0	37.3	24.0	2.2	25.4	18.0	2.1	0.79	**0.006**	**0.01**
No. of Wins	4.0	2.0	0.3	4.2	2.0	0.4	1.9	1.0	0.4	0.77	**0.008**	**0.01**
No. of Placings	10.9	6.0	0.8	11.6	7.0	0.9	6.5	3.0	0.8	0.12	0.21	**0.03**
Wins (freq.)	0.10	0.06	0.01	0.10	0.08	0.01	0.06	0.03	0.01	0.28	0.99	0.49
Placings (freq.)	0.24	0.23	0.01	0.28	0.27	0.01	0.22	0.19	0.02	0.45	0.89	0.73
Earnings (SEK)^c^	223,600	86,150	23,052	259,000	101,400	30,785	104,700	42,290	17,051	0.07	0.43	**0.05**
Earnings/start (SEK)^c^	4609	3130	387	5224	3944	344	3162	2594	242	0.13	**0.03**	**0.002**
Time record volstart (sec/km)	90.8	90.4	0.3	90.5	90.0	0.3	92.5	91.5	0.4	**0.05**	0.38	**0.04**
Time record autostart (sec/km)	89.0	89.0	0.2	88.8	88.6	0.2	89.8	89.9	0.3	0.34	0.25	0.10

a Transformed values were used for the analysis: log_10_, ln(earnings + 1 000) and ln(race time − 68.2).

b Linear model analyses were performed in R. Significant results (*P *≤* *0.05) in bold.

c SEK, Swedish Kronor.

### Haplotype analyses

Haplotype analysis was performed using 7 SNPs, including the 5 SNPs that were significantly associated with racing performance and two additional closely located SNPs (Table 9). Four haplotypes were present in the population. The TGTAAAG haplotype was the most common (0.34) and it was nominated as the base haplotype. The haplotype TTCGGGA was significantly associated with number of starts and number of wins (*P *≤* *0.05, Table 9). None of the other haplotypes showed significant associations with racing performance.

**Table 9:** Haplotype frequencies, haplotype coefficients, and *P*‐values for the Coldblooded trotter performance results

	Haplotype 1^a^		Haplotype 2		Haplotype 3		Haplotype 4	
Haplotype^b^	TGTAAAG		GGTAAAA		TTCGGGA		GTCGGGG	
Frequency	0.34		0.33		0.19		0.12	
Performance trait	Haplotype coefficient	*P*‐value	Haplotype coefficient	*P*‐value^c^	Haplotype coefficient	*P*‐value^c^	Haplotype coefficient	*P*‐value^c^
No. of starts	–	–	<0.01	0.95	−0.10	**0.05**	−0.07	0.22
No. of wins	–	–	−0.02	0.41	−0.08	**0.01**	−0.03	0.38
Wins (freq.)	–	–	−0.05	0.28	−0.03	0.60	−0.05	0.44
No. of placings	–	–	0.03	0.17	<−0.01	0.90	0.03	0.22
Placings (freq.)	–	–	0.04	0.12	0.03	0.32	0.04	0.25
Earnings (SEK)^d^	–	–	−0.09	0.16	−0.04	0.63	−0.03	0.73
Earnings/start (SEK)^d^	–	–	−0.07	0.21	−0.09	0.19	−0.10	0.21
Time record voltstart (sec/km)	–	–	<0.01	0.36	<0.01	0.76	<0.01	0.72
Time record autostart (sec/km)	–	–	<0.01	0.93	<−0.01	0.72	0.02	0.21

a Order of the SNPs: Chr22: 45,732,929; 45,748,082; 45,748,491; 45,748,586; 45,749,526; 45,749,595; 45,752,522.

b The most common haplotype (TGTAAAG) was used as the base haplotype.

c A GLM regression analysis was performed in R. Significant results (*P *≤* *0.05) in bold.

d SEK, Swedish Kronor.

### Part iii: Genotyping of the highest ranked SNP in 18 different horse breeds

The genotype frequencies for the SNP on chromosome 22 in 18 different breeds are presented in Table 10.

**Table 10:** Allele frequencies for the SNP g.22:45748491C>T in different horse breeds^a^

Breed	*n*	T	C
Arabian Thoroughbred	91	1.00	0.00
Thoroughbred^b^	91	0.99	0.01
Shire horse	30	0.98	0.02
Swedish Warmblood	77	0.96	0.04
Quarter horse	40	0.93	0.08
American Curly	87	0.91	0.09
Standardbred	250	0.86	0.14
Coldblooded trotter	183	0.70	0.30
American Miniature	14	0.61	0.39
Finnhorse	157	0.61	0.39
Shetland‐ and minishetland	104	0.42	0.58
Icelandic horse	167	0.26	0.74
Ardennes	47	0.20	0.80
Gotlandsruss	153	0.19	0.81
North‐Swedish draught horse	53	0.10	0.90
Exmoor^b^	271	0.02	0.98
Fjordhorse	50	0.01	0.99
Haflinger	50	0.01	0.99

a Horses were genotyped using the StepOnePlus Real‐Time PCR System TaqMan.

b Genotype data was obtained from the 670K Affymetrix Equine Genotyping Array (Exmoor pony) or the Illumina SNP70 Genotyping BeadChip (Thoroughbred).

## Discussion

By comparing the genomes of Coldblooded trotters, North‐Swedish draught horses, and Standardbreds we identified five genetic regions, where the trotters were similar to each other but different to the North‐Swedish draught horses. The window with the highest Delta Fst value was located about 50 kb from the *EDN3* gene. In total five SNPs in the region demonstrated significant associations with racing performance traits in Coldblooded trotters. The haplotype association analysis also revealed significant negative associations between number of starts and number of wins and the haplotype TTCGGGA (Table 9). The SNP association analysis indicated a dominant inheritance effect of the haplotype associated with the identified top SNP, because the CC genotype but not the CT genotype was negatively correlated with performance (Table 3). Interestingly, there was a high frequency of the T‐allele in high‐performance breeds such as Thoroughbreds, Standardbreds, Quarter horses, Coldblooded trotters, and Swedish Warmbloods, while the pony breeds and draught horses, that is, Exmoor, Shetland ponies, Gotlandsruss, Ardennes, and the North‐Swedish draught horses, all displayed a high frequency of the CC genotype (Table 10).

While most of the breeds that displayed a high frequency of the TT genotype are traditionally used for different types of racing or sports, it is worth noting that the frequency of the TT genotype was high also in American Miniature, American Curly, and Shire horses, three breeds not typically used for racing or jumping competitions. Likely, the most significantly associated SNP identified and analyzed in the current study is not a causative mutation, and further studies are required to fully understand the impact of the region identified and which mutation that is the causative variant. Given the genetic distance between the region and the *EDN3* gene it is possible that the region includes a regulatory element that either influences the expression *EDN3* or any of the other genes in that location. The EDN3 protein is a member of the endothelin family and the active form of the protein is a 21 amino acid vasoactive peptide (Yanagisawa et al. 1988; Inoue et al. 1989). The protein is a ligand that binds to the endothelin receptor type B (EDNRB) (Baynash et al. 1994; Hosoda et al. 1994). The binding of the ligand to the receptor is crucial for the development of melanocytes and enteric neurons, and mutations in *EDN3* and *EDNRB* have been associated with congenital disorders such as Hirschprung disease and Waardenburg syndrome in humans and lethal white foal syndrome (LWFS) in horses (Baynash et al. 1994; Hosoda et al. 1994; Puffenberger et al. 1994; Edery et al. 1996; Hofstra et al. 1996; Kusafuka et al. 1997; Metallinos et al. 1998; Santschi et al. 1998; Yang et al. 1998; Lee et al. 2003; Stanchina et al. 2006). While no previous studies have reported any associations between the *EDN3* gene and performance in horses, it is possible that the gene influences performance by regulating blood supply. In humans, the gene has been associated with variations in blood pressure and cardiovascular disease risk (Levy et al. 2009; International Consortium for Blood Pressure Genome‐Wide Association Studies, 2011). For a horse to perform at a high level the distribution of blood to the tissues is crucial, and any disruptions to blood flow can have major impacts on performance (Evans 2007). A low stroke volume may limit the maximal cardiac output, which will affect the energy output negatively (Evans 2007). Interestingly, a previous study has observed an increase in the concentration of the related protein EDN1 after exercise in horses, and another study suggested a plausible contribution of *EDN1* to the pathogenesis of asthma in horses (Benamou et al. 1998; McKeever et al. 2002).

**Figure 2:**
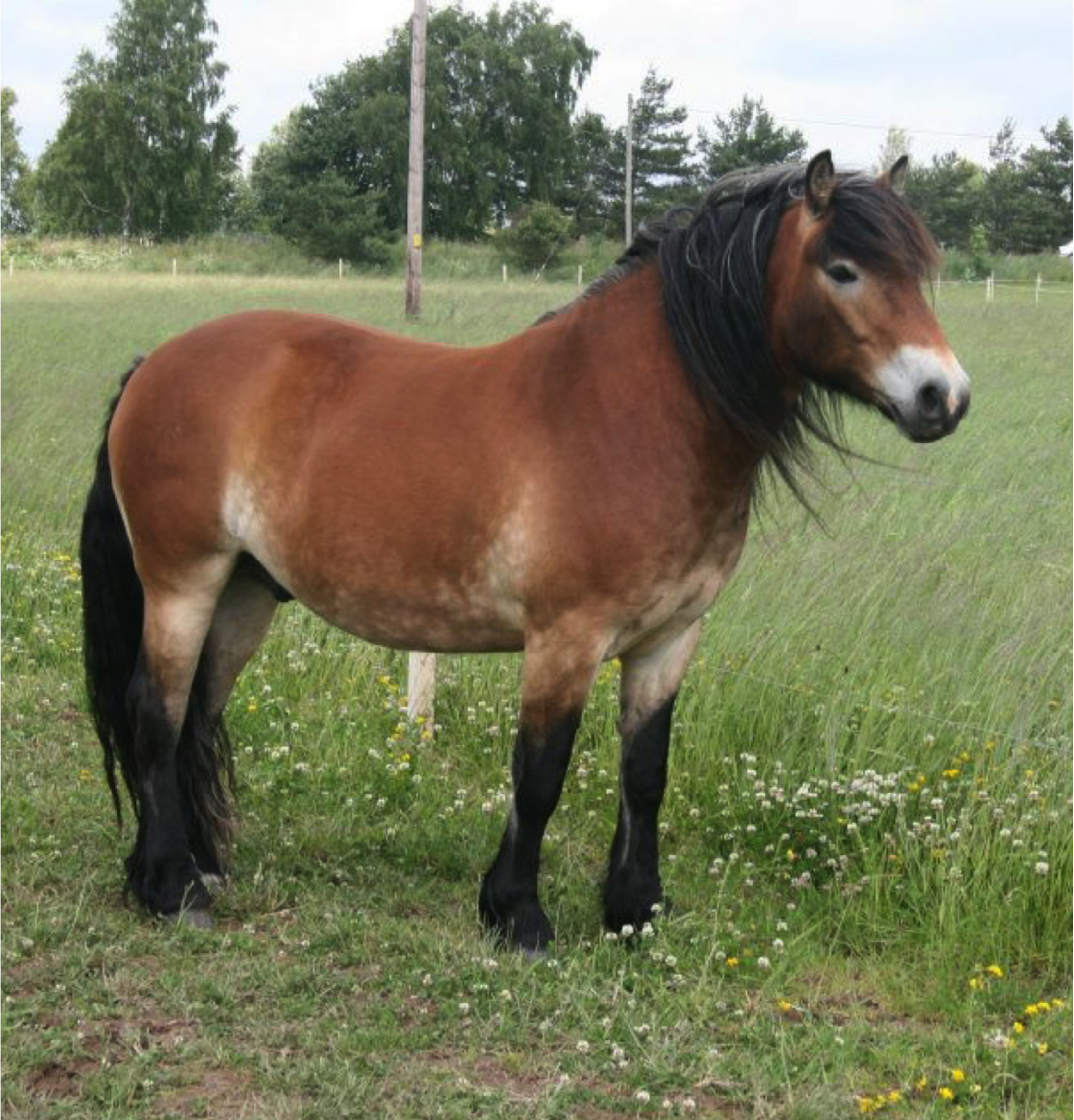
A North‐Swedish draught horse with the “mealy” coat color. Photo: Renée Adolphsson.

**Figure 3:**
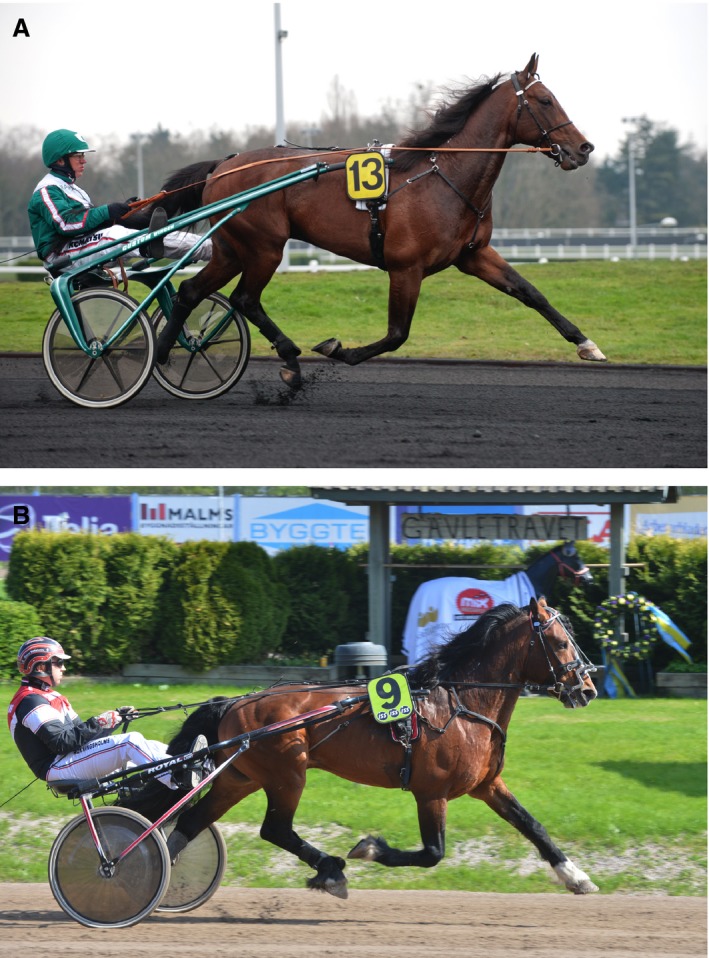
(A) Standardbred trotter. Photo: Kanal 75 AB, Sweden. (B) A Coldblooded trotter. Photo: Kanal 75 AB, Sweden.

Given the known role of *EDN3* and *EDNRB* for pigmentation development and coat color it is interesting to note the difference in color that occurs between Coldblooded trotters, Standardbreds and North‐Swedish draught horses (Hosoda et al. 1994; Puffenberger et al. 1994). While many draught horses display a coat color referred to as “pangare” or “mealy”, that is, a light muzzle and often a light belly (Figure 2), few Coldblooded trotters and almost no Standardbreds show this color (Figure 3) (Sponenberg 2009). In breeds where all horses display the mealy phenotype, such as Exmoor and Fjordhorses, horses also appear to be predominantly fixed for the C‐allele (Table 10). This suggests a possible link between the region identified and coat color and supports the possibility that we have identified a locus that disrupt pigmentation. However, the mutation may have pleiotropic effects, as the current study demonstrated a significant association of the SNPs with harness racing performance. This theory is supported by the fact that while it is common in some horse breeds, trotters are not selected for coat color but solely for performance related traits.

Aside from the *EDN3* region, none of the SNPs in the windows identified the Delta Fst analysis were associated with racing performance in Coldblooded trotters. None of the previously reported performance genes were identified in the top windows (Gu et al. 2009, 2010; Binns et al. 2010; Hill et al. 2010a,b; Schröder et al. 2011; Andersson et al. 2012; Thomas et al. 2014). That includes for example the *DMRT3* and Myostatin (*MSTN*) genes, which have been reported to have major influence on performance in harness racing breeds and Thoroughbreds (Binns et al. 2010; Hill et al. 2010a; Andersson et al. 2012). It is possible that the Delta Fst comparison identified regions/genes that are important for other traits that differ between the breeds, for example morphological traits, given the differences in morphology that exists between the breeds (Figs. 2, 3). It could also be that the effects of the SNPs analyzed were too small to accurately detect in the analysis. As only five regions were investigated for association with performance, it is likely that more regions from the Delta Fst analysis may be associated with performance. Additionally, the low number of significant performance associations may be due to the small sample size in the Delta Fst analysis or low genetic variation for the SNPs analyzed. After filtering and quality assurance the horses were analyzed for approximately 37,000 SNPs, which gives a sparse coverage of the genome with an average distance of 72.5 kb between the markers. The Fst was calculated for windows of 5 SNPs and the windows identified were rather large. It is possible that some SNPs with a high single Delta Fst value were down weighted by the neighboring SNPs and therefore not discovered in this analysis. For future studies we will address this issue using whole‐genome sequence data and including a larger number of horses. By doing so we aim to not only confirm the regions identified in this study but also to identify additional regions that may be of importance for racing performance. Furthermore studies of the potential role of *EDN3* for performance and exercise in horses are necessary to understand how this gene may contribute to racing performance. Nevertheless, the results from this study provide important information about the complex genetic regulation of performance with additional studies needed to fully understand the exact impact of the identified region on harness racing performance.

## Conclusions

From the Delta Fst analysis five genomic regions where Coldblooded trotters and Standardbreds were genetically similar but together differed from North‐Swedish draught horses were identified and used for association analysis with harness racing performance. One SNP, g.22:45748491C>T, was significantly associated with racing performance results in 400 Coldblooded trotters. The CC genotype appeared to negatively influence performance results. Interestingly, the TT genotype was at a high frequency in athletic breeds such as Thoroughbreds, Standardbreds, Coldblooded trotters, and Warmbloods while the frequency of the CC genotype was high in pony breeds and draught horses. The closest gene next to the SNP identified was the *EDN3* gene, which encodes for a vasoactive peptide that is crucial for the development of melanocytes and enteric neurons. The study provides novel information about the genetics of horse performance, but additional studies are needed to confirm the findings.

## Conflict of Interest

There are no conflicts of interest to declare.
